# DSMRI: Domain Shift Analyzer for Multi-Center MRI Datasets

**DOI:** 10.3390/diagnostics13182947

**Published:** 2023-09-14

**Authors:** Rafsanjany Kushol, Alan H. Wilman, Sanjay Kalra, Yee-Hong Yang

**Affiliations:** 1 Department of Computing Science, University of Alberta, Edmonton, AB T6G 2R3, Canada; 2 Departments of Radiology and Diagnostic Imaging and Biomedical Engineering, University of Alberta, Edmonton, AB T6G 2R3, Canada; 3 Division of Neurology, Department of Medicine, University of Alberta, Edmonton, AB T6G 2R3, Canada

**Keywords:** MRI, domain shift, t-SNE, UMAP, quality control, texture analysis

## Abstract

In medical research and clinical applications, the utilization of MRI datasets from multiple centers has become increasingly prevalent. However, inherent variability between these centers presents challenges due to domain shift, which can impact the quality and reliability of the analysis. Regrettably, the absence of adequate tools for domain shift analysis hinders the development and validation of domain adaptation and harmonization techniques. To address this issue, this paper presents a novel *Domain Shift analyzer for MRI* (DSMRI) framework designed explicitly for domain shift analysis in multi-center MRI datasets. The proposed model assesses the degree of domain shift within an MRI dataset by leveraging various MRI-quality-related metrics derived from the spatial domain. DSMRI also incorporates features from the frequency domain to capture low- and high-frequency information about the image. It further includes the wavelet domain features by effectively measuring the sparsity and energy present in the wavelet coefficients. Furthermore, DSMRI introduces several texture features, thereby enhancing the robustness of the domain shift analysis process. The proposed framework includes visualization techniques such as t-SNE and UMAP to demonstrate that similar data are grouped closely while dissimilar data are in separate clusters. Additionally, quantitative analysis is used to measure the domain shift distance, domain classification accuracy, and the ranking of significant features. The effectiveness of the proposed approach is demonstrated using experimental evaluations on seven large-scale multi-site neuroimaging datasets.

## 1. Introduction

The development of magnetic resonance imaging (MRI), which offers non-invasive examination of the human brain’s structure and composition, has revolutionized medical imaging. High-resolution images of brain anatomy are obtained during MRI scans, allowing medical professionals and researchers to examine different neuroanatomical aspects like cerebral structures, white matter, and grey matter. MRI datasets play a vital role in advancing medical research, not only in aiding in the understanding, diagnosing, and treating of numerous neurological disorders but also in training deep learning models. Radiologists and neurologists use MRI scans to identify abnormalities, such as tumours, lesions, atrophy, or other anatomical changes, that may be signs of disorders like Alzheimer’s, multiple sclerosis, epilepsy, and brain tumours [1,2,3]. In recent years, the availability of large-scale multi-center datasets has significantly advanced medical imaging research, which opens the avenues for developing powerful machine learning (ML) algorithms and data-driven methodologies.

Multi-center MRI datasets, incorporating data from multiple imaging centers or institutions, offer a unique opportunity to leverage diverse patient demographics, equipment, imaging platforms, and protocols. These datasets are valuable resources that can improve the generalizability and representativeness of research, producing more robust and reliable findings. Additionally, multi-site databases make it easier to investigate rare disorders, assess novel imaging techniques, and establish clinical benchmarks [4]. However, despite their potential advantages, multi-center MRI datasets introduce significant challenges due to a phenomenon known as *“domain shift"* [5]. Domain shift is the term used to describe the variances in data distributions across different centers resulting from variations in hardware, acquisition protocols, patient demographics, and environmental factors. These distributional shifts can severely affect the performance and generalizability of ML strategies and analysis techniques trained in one center and applied to another.

In a multi-center MRI dataset, domain shift primarily arises due to the heterogeneity among MRI scanners and imaging protocols across different centers. Some examples of domain shift parameters include imaging protocol (flip angle, acquisition orientation, slice thickness, and resolution) and scanner (manufacturer, model, magnetic field strength, and number of channels per coil). As a result, the appearance, contrast, intensity distributions, spatial resolutions, and noise levels of MR images differ qualitatively and quantitatively from site to site and study to study.

The problem of domain shift creates several challenges in analyzing and interpreting multi-center MRI datasets. Firstly, it impacts the performance and reliability of ML analysis pipelines as models trained in one center may fail to generalize effectively to the data from other centers [6]. This issue can hinder the adoption of automated tools for diagnosis, treatment planning, and disease monitoring as their efficacy relies on their capability to handle data from diverse sources. Secondly, domain shift can introduce biases and confounds in research studies that utilize multi-site MRI datasets. In clinical trials or population studies involving data from multiple centers, the variations originating from domain shift might distort statistical analysis, leading to erroneous conclusions and misleading findings. Thirdly, the inherent variability in scanner hardware and software across centers can introduce technical discrepancies, further complicating the comparison and fusion of data. These issues pose significant challenges for researchers and clinicians seeking to extract reliable and reproducible insights from multi-center MRI datasets.

Addressing the challenges of domain shift in multi-site MRI datasets requires advanced techniques and methodologies. Domain adaptation (DA) [7,8] and harmonization [9] methods aim to bridge the gap among different domains by aligning and normalizing the data from different centers. These approaches involve transforming the data distribution or features to minimize domain-specific variations, enabling more bias-free and reliable analysis across centers. Before developing DA or harmonization algorithms, it is essential to comprehensively understand the nature of domain shift in existing or target datasets. The degree of domain shift in a multi-center MRI dataset is a problem worth investigating and is the principal focus of this article. In particular, we propose a novel framework named DSMRI (Domain Shift analyzer for MRI) to qualitatively and quantitatively determine the degree of domain shift present in an MRI dataset. The proposed framework leverages existing MRI-quality-related spatial domain features as well as introduces frequency, wavelet, and texture domain features to quantify the degree of domain shift. To assess the effectiveness of the proposed framework, we conduct comprehensive experiments with seven multi-site MRI datasets, including participants with amyotrophic lateral sclerosis (ALS), Alzheimer’s disease (AD), Parkinson’s disease (PD), and autism spectrum disorder (ASD) in addition to healthy controls (HC). To foster reproducibility and knowledge sharing, the Python source code of the DSMRI has been made publicly available at https://github.com/rkushol/DSMRI (accessed on 5 September 2023). The applications and benefits of analyzing and dealing with domain shift in multi-center MRI datasets are numerous. Here are some crucial ones:

Improved generalizability: Domain shift analysis facilitates the development of ML models that can generalize across multiple centers. By identifying and mitigating the variations caused by domain shift, the methods become more robust and applicable to data from different imaging centers.

Reliable and reproducible research: It helps overcome biases and confounds triggered by the variations across different sites. By accounting for the domain-specific effects, research studies utilizing multi-center MRI datasets can yield more reliable and reproducible results.

Cross-center comparison and validation: It enables meaningful comparisons and validation of imaging biomarkers, algorithms, and protocols across various centers. Thus, researchers and clinicians can assess the performance and consistency of imaging techniques and analysis methods in diverse settings.

Enhanced collaborative research: Multi-center collaborations have become prevalent in medical imaging research. Analyzing domain shift encourages data sharing and collaboration among different centers by enabling a harmonized data analysis from various sources. It promotes data integration, pooling, and joint analysis, thereby facilitating large-scale studies and advancing scientific knowledge in the field.

Adaptation to new centers and populations: As new imaging centers are established, or new patient cohorts are included in studies, domain shift analysis can guide the adaptation of existing models to these new domain configurations. This reduces the time and effort required to deploy analysis tools in new settings, allowing faster translation of research findings into clinical practice.

Quality control (QC) and outlier detection: Analyzing domain shift can serve as a QC measure for MRI datasets. It allows for identifying centers or specific scans that exhibit significant variations compared to others. Such insights can help in data validation as well as detect potential sources of errors or outliers.

The proposed DSMRI framework, explicitly designed to analyze the presence of domain shift in multi-center MRI datasets, to our best knowledge, offers several significant contributions for the first time. Firstly, DSMRI integrates insights from diverse domains, including spatial, frequency, wavelet, and texture analysis. This multi-domain approach fortifies the framework’s ability to capture various aspects of domain shift. Secondly, deriving the features from the frequency domain to capture low- and high-frequency image information and incorporating wavelet domain features to measure sparsity and energy within wavelet coefficients enhance the robustness of domain shift analysis. Thirdly, using visualization techniques such as t-SNE [10] and UMAP [11] enriches the framework’s ability to visually represent and interpret domain shift effects. Fourthly, estimating domain shift distance, domain classification accuracy, and the ranking of significant features adds a rigorous quantitative evaluation of domain shift. Lastly, the efficacy of DSMRI is validated through extensive experimental evaluations conducted on seven large-scale multi-site neuroimaging datasets. This real-world validation showcases the practical applicability of the proposed framework.

## 2. Related Works

### 2.1. Domain Shift in Multi-Center MRI Datasets

Prior studies have widely acknowledged and examined the presence of domain shift in multi-center MRI datasets. Researchers have consistently reported variations and challenges originating from domain shift, highlighting the need for robust analysis techniques.

A study by Dadar et al. [12] examined the impact of scanner manufacturers on a brain MRI dataset collected from multiple imaging centers. They reported significant differences in grey and white matter volume estimation among scanner manufacturers. These variations affected the reliability of automated brain segmentation algorithms, resulting in inconsistent outcomes from different centers. In another investigation by Tian et al. [13], domain shift effects were analyzed to reduce the site effects on grey matter volume maps using a travelling-subject MRI dataset obtained from various sites. They considered several underlying domain shift factors, such as scanner manufacturer, model, phase encoding direction, and channels per coil. Interestingly, they found that the scanner manufacturer is the most significant parameter causing domain shift, followed by the scanner model.

In another study, Lee et al. [14] explored the effects of changing MRI scanners on whole-brain volume change estimation at different time point visits. They identified that inter-vendor (e.g., Philips to Siemens) scanner changes led to more significant effects on percentage brain volume change than intra-vendor (e.g., GE Signa Excite to GE Signa HDx) scanner upgrades. Additionally, Glocker et al. [15] conducted an empirical study to investigate the impact of scanner effects when using ML on multi-site neuroimaging data. The authors discovered that, even after meticulous pre-processing using advanced neuroimaging tools, a classifier could identify the origin of the data (e.g., scanner) with very high accuracy. Moreover, Panman et al. [16] experimented with eight-channel and thirty-two-channel head coil configurations using structural, diffusion, and functional MR images while keeping all other parameters identical. They showed that the variations in the number of head coils could considerably impact the outcomes of analysis methods despite having the acquisition parameters synchronized.

The above studies collectively highlight the pervasive presence of domain shift in multi-center MRI datasets. The observed variations in image characteristics and acquisition parameters across centers pose considerable challenges for analysis and interpretation.

### 2.2. Quality Assessment Methods for MRI Data

MRIQC [17] is an open-source tool developed to automatically predict the quality of MRI data acquired from unseen sites as manual inspection is subjective and impractical for large-scale datasets. The tool extracts a set of spatial domain features to train an ML classifier and predict whether a scan should be accepted or excluded from the analysis. The authors validated that MRIQC accurately predicted image quality on an unseen dataset of multiple scanners and sites with approximately 76% accuracy. To address the errors and inconsistencies in brain image segmentation, Mindcontrol [18], a web-based application, was designed to allow a user to inspect brain segmentation data and manually correct errors visually. The user can view and interact with 3D brain images, including the ability to adjust opacity, slice orientation, and zoom level for data curation and QC.

Osadebey et al. [19] presented a quality metric scheme for structural MRI data in multi-site neuroimaging studies. The system evaluates image quality based on factors such as luminance contrast, texture analysis, and lightness and generates a total quality score. The authors demonstrated the system’s effectiveness by applying it to large-scale multi-center MRI data and concluded that it correlates well with human visual judgment. The quality evaluation using multi-directional filters for MRI (QEMDIM) [20] is a technique that is capable of detecting various distortions, including Gaussian noise and motion artifacts. The method utilizes mean-subtracted contrast-normalized (MSCN) coefficients to extract image statistics in the spatial domain. Their evaluation showed satisfactory accuracy in identifying low-quality images affected by different artifacts or noises compared to undistorted images.

Esteban et al. [21] proposed a crowdsourcing approach for collecting MRI quality metrics and expert quality annotations to train both humans and machines in assessing the quality of MRI data. They revealed that the ML algorithms trained on the crowdsourced data perform comparably to human raters in evaluating image quality. The strategy developed by Oszust et al. [22], NOMRIQA, applies high-boost filtering to intensify the high-frequency points, which allows the identification of various distortions. Their method utilizes the fast retina key-point descriptor and the support vector regression classifier to generate a quality score, which assists in detecting distorted T2-weighted images.

Bottani et al. [23] introduced an automated QC method for brain T1-weighted MRI in a clinical data warehouse. The technique involves extracting spatial domain features using a convolutional neural network (CNN) to predict scans that need to be excluded. They showed that their method could recognize images with potential quality issues, such as artifacts or motion-related distortions, and detect acquisitions for which gadolinium was injected. Lastly, an overview of various no-reference image quality assessment (NR-IQA) methods designed explicitly for MRI can be found here [24]. The authors discussed the challenges associated with evaluating MRI image quality due to the complex and dynamic nature of MRI data, including the influence of various acquisition parameters, image artifacts, and population-related factors.

These QC studies focus mainly on automatically detecting artifacts or poor-quality samples to reduce manual effort and decide whether a particular scan should be accepted or excluded from the analysis. These studies neither emphasize quantifying the degree of domain shift from these QC features nor analyze which features are correlated to domain shift.

### 2.3. Existing Domain Shift Analysis Tools

The tools introduced by Sadri et al., MRQy [25], and Guan et al., DomainATM [26], can be considered the two closest studies related to the proposed framework. MRQy is mainly designed for the QC of MRI data by which manual effort to filter poor-quality data can be automated for clinical and research studies. It uses different spatial-domain-image-quality-related metrics to address different types of noise, shading, inhomogeneity, and motion artifacts. Although they provided an example of detecting site effects using their proposed features, when we experimented on large-scale datasets with more scanner/acquisition protocol variations, we noticed that MRQy features could not cluster the data accurately. Secondly, MRQy uses metadata, such as image/voxel dimension from the file header. These features become identical for all the center’s data after commonly used preprocessing steps like skull stripping or registration; hence, they are not fruitful for site effect analysis.

DomainATM offers visualization of data distribution as well as measures the domain shift distance for the original or synthetic data. Then, they implemented some classical DA methods to show the effectiveness of these methods in reducing the domain shift. However, this tool cannot take raw neuroimaging data, such as NIfTI files, directly as input. To analyze real-world data with DomainATM, the user must process the data with Anatomical Automatic Labeling (AAL) atlas and then extract the grey matter volumes for each region of interest (ROI), making the tool inconvenient for many applications. Most importantly, these grey matter features are not meaningful regarding the domain shift measurement, which is reflected in the experimental section. The proposed framework DSMRI is compared with MRQy and DomainATM to demonstrate the strength of the proposed features in analyzing the domain shift in a multi-center MRI dataset.

## 3. Materials and Methods

### 3.1. Datasets

Seven large-scale multi-center datasets are used in the experimental evaluation of the proposed framework. Publicly available Alzheimer’s Disease Neuroimaging Initiative (ADNI) [27] and the Australian Imaging, Biomarker and Lifestyle (AIBL) [28] datasets comprise AD patients and HC. The Parkinson’s Progression Markers Initiative (PPMI) [29] and the Autism Brain Imaging Data Exchange (ABIDE) [30] are also publicly available datasets containing MRI data with PD and ASD patients. The Canadian ALS Neuroimaging Consortium (CALSNIC) [31] multi-site dataset incorporates ALS patients along with HC. For ADNI and CALSNIC, two independent versions are used, ADNI1/ADNI2 and CALSNIC1/CALSNIC2, respectively. The T1-weighted structural MR images are used for all seven databases. Furthermore, we evaluate the outcomes for the T2-weighted and FLAIR (Fluid Attenuated Inversion Recovery) images of the CALSNIC2 dataset. All the aforementioned datasets comprise data from three widely used scanner manufacturers (GE Healthcare, Philips Medical Systems, and Siemens) except the AIBL, which only includes Siemens vendor data. Table 1 and Supplementary Table S1 illustrate each dataset’s demographics and scanning details, respectively.

### 3.2. Proposed Features

**Figure 1:**
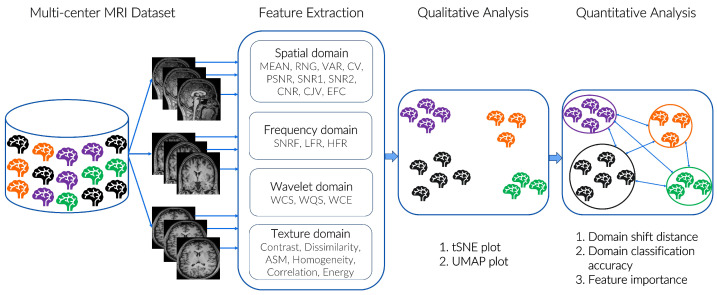
An overview of the proposed DSMRI framework. The different colours in the brain icon show that MRI data originated from different sites or may be acquired with distinct image acquisition protocols. Twenty-two significant features are extracted from 2D MRI slices of each subject. Utilizing these feature maps, t-SNE and UMAP methods are used to visualize the position of each scan in a reduced two-dimensional plot. The results are also interpreted in quantitative analysis, where the domain shift distance can be obtained with the maximum mean discrepancy distance (MMD) and the ranking of 22 features to show which features play a more significant role in classifying different domains. Best viewed in colour.

We leverage various image-quality-related metrics to quantify the degree of domain shift in a multi-center MRI dataset. An overview of the proposed DSMRI framework is shown in Figure 1. The 22 features used in the proposed framework are summarized in Table 2. The features are extracted from the foreground of 2D slices of 3D MRI in three different directions, i.e., axial, sagittal, and coronal. MRQy is used to detect the foreground of the MR image. However, Signal-to-Noise Ratio (SNR), Contrast-to-Noise Ratio (CNR), and Coefficient of Joint Variation (CJV) features also involve the background intensity information to measure their corresponding quality score.

#### 3.2.1. Spatial Domain Features

Spatial domain features are based on prior studies [17,25,32,33,34]. Firstly, essential statistical intensity distributions of the foreground (F) are extracted by $MEAN=\frac{1}{HW}\sum_{i,j}F\left(i,j\right)$, Range (RNG)=max(F)−min(F), and Variance $\left(VAR\right)=\sigma_{F}^{2}$, where H=height,W=width, and $\sigma^{2}=variance$.

Secondly, relevant noise-related features are extracted by incorporating F and the background (B), such as Peak SNR (PSNR) following [25], $SNR1=\frac{\sigma_{F}}{\sigma_{B}}$, $SNR2=\frac{\mu_{FP}}{\sigma_{B}}$, and $CNR=\frac{\mu_{FP-BP}}{\sigma_{B}P}$, where μ= mean, σ= standard deviation (SD), FP= Foreground Patch, and BP= Background Patch. FP and BP are random 5×5 square patches of *F* and *B*, respectively.

Finally, to detect different types of artifacts like shadowing, inhomogeneity, aliasing, and motion, we employ Coefficient of Variation (CV), CJV, and Entropy Focus Criterion (EFC) features extracted from F. The CV is defined as $CV=\frac{\sigma_{F}}{\mu_{F}}$, whereas the CJV can be expressed as $CJV=\frac{\left(\sigma_{F}+\sigma_{B}\right)}{|\mu_{F}-\mu_{B}|}$. The EFC is defined as $EFC=\frac{HW}{\sqrt{HW}}log\frac{E}{\sqrt{HW}}$, where *E* is derived following [25].

#### 3.2.2. Frequency Domain Features

The frequency domain features are calculated after performing a 2D Fast Fourier Transform (FFT) on F. The FFT is performed with the Python SciPy library [35].

1. SNRF: The SNR in the frequency domain assesses signal quality corrupted by noise. It quantifies the power (or energy) ratio in the signal component to the power (or energy) in the noise component in the frequency domain. It can be defined as $SNRF=10*log(\frac{P_{signal}}{P_{noise}})$, where $P_{signal}$ is the power (or energy) in the signal component and $P_{noise}$ is the power (or energy) in the noise component.

2. LFR: The Low Frequency Response (LFR) has the ability to capture low-frequency information in the resulting image. It involves applying a low-pass filter, calculating the amplitude spectrum using the FFT, and measuring the LFR as the square root of the amplitude spectrum. The purpose of the low-pass filter is to attenuate or remove high-frequency components from F, allowing only low-frequency information to pass through. A 3×3 Gaussian filter [[1,2,1],[2,4,2],[1,2,1]]/16 is used as a low-pass filter, which is convolved with F to obtain a low-pass version of F. The amplitude spectrum of the low-pass image is then computed using the FFT, representing the distribution of frequencies present in the low-pass image. Finally, the square root operation is performed to linearize the amplitude spectrum and make it more suitable for interpretation. The LFR can be expressed as follows: $LFR=\sqrt{FFT(\mathrm{low}\_\mathrm{pass}\_\mathrm{image})}$.

3. HFR: Similar to the concept of LFR, the High Frequency Response (HFR) has the ability to capture high-frequency information in an image. Instead of a low-pass filter, a high-pass filter is applied to F, allowing only high-frequency components to pass through while attenuating lower frequencies. This step emphasizes the high-frequency content of an image. A 3×3 Laplacian filter [[−1,−1,−1],[−1,8,−1],[−1,−1,−1]] is used as a high-pass filter, which is convolved with F to acquire a high-pass version of F. After applying FFT to the high-pass image, the final HFR can be measured as follows: $HFR=\sqrt{FFT(\mathrm{high}\_\mathrm{pass}\_\mathrm{image})}$.

#### 3.2.3. Wavelet Domain Features

The wavelet domain features are extracted after performing a 2D discrete wavelet transform (DWT) on F. The wavelet decomposition is implemented with the Python Pywt package [36], and some examples of wavelet types include Haar, Daubechies, Discrete Meyer, Symlets, and Coiflets.

1. WCS: The Wavelet Coefficient Sparsity (WCS) is a feature used to measure the amount of sparse information present in the wavelet coefficients of a signal or an image. It quantifies the extent to which the wavelet coefficients are concentrated in a few significant coefficients while the majority are close to zero or negligible. First, a 2D DWT is applied to F, which decomposes an image into different frequency subbands, representing different scales or levels of detail. Then, the wavelet coefficients obtained from the wavelet transform are analyzed to determine their sparsity. Various sparsity measurement techniques can be employed, such as counting the number of coefficients above a certain threshold or using sparse representation algorithms like l1-norm minimization. Here, the WCS is measured based on the mean of the coefficients. Coefficients above the mean are considered significant, while those below the mean are considered insignificant. The WCS can be represented as follows: $WCS=\frac{\sum_{i}^{n}\left|\mathrm{Significant}\_{\mathrm{Coefficient}}_{i}\right|}{n}$.

2. WQS: The Wavelet-based Quality Score (WQS) evaluates the quality of an image by analyzing its spatial frequency content using the wavelet transform. It calculates a quality score based on the magnitude and phase information of the wavelet coefficients. The magnitude represents the strength or energy of each coefficient, while the phase represents the spatial orientation or phase shift. The WQS is calculated by taking the sum of the product of magnitude and cosine form of the phase for each coefficient. The WQS can be expressed as follows: $WQS=\sum_{i}^{n}\left({\mathrm{magnitude}}_{i}*cos\left({\mathrm{phase}}_{i}\right)\right)$, where ${\mathrm{magnitude}}_{i}=\left|{\mathrm{Coefficient}}_{i}\right|$ and ${\mathrm{phase}}_{i}=angle\left({\mathrm{Coefficient}}_{i}\right)$.

3. WCE: The Wavelet Coefficient Energy (WCE) measures the amount of energy present in the wavelet coefficients of a signal or an image. It quantifies the overall strength or magnitude of the coefficients, indicating how much information is contained in each coefficient. The energy of each wavelet coefficient is computed by taking the absolute value of its magnitude. The total energy of the wavelet coefficients is then obtained by summing up the energies of all the coefficients. The WCE can be defined as follows: $WCE=\frac{\sum_{i}^{n}\left|{\mathrm{Coefficient}}_{i}\right|}{n}$.

#### 3.2.4. Texture Domain Features

Texture features are extracted from the widely used GLCM [37,38], which represents the spatial relationship between pairs of pixel intensities in an image. These features provide valuable information about the spatial patterns and structures present in an image, enabling the characterization and differentiation of various textures within an image. We employ the Python scikit-image package [39], which provides a convenient way to calculate various GLCM texture features. A brief description of six GLCM features employed in the proposed framework is given below:

1. Contrast: This feature measures the local variations or differences in intensity between neighbouring pixels in an image. It provides information about the amount of contrast present in the image texture. It is calculated as the sum of squared intensity differences between neighbouring pixel pairs, weighted by the frequencies in the GLCM matrix. A higher contrast value indicates greater variation or sharp transitions between pixel intensities, representing a more textured or detailed image. Command: skimage.feature.greycoprops(GLCM, ‘contrast’). Formula:

(1) $$\begin{array}{c} \mathrm{Contrast}=\sum_{i,j}{\left(i-j\right)}^{2}\cdot \mathrm{GLCM}\left(i,j\right) \end{array}$$

2. Dissimilarity: This feature calculates the average absolute difference between the pixel intensities in the GLCM. It quantifies the amount of local variation in the texture. Higher values indicate greater pixel dissimilarity. It is similar to contrast but focuses on absolute differences rather than squared differences. Command: skimage.feature. greycoprops (GLCM, ‘dissimilarity’). Formula:

(2) $$\begin{array}{c} \mathrm{Dissimilarity}=\sum_{i,j}\left|i-j\right|\cdot \mathrm{GLCM}\left(i,j\right) \end{array}$$

3. Angular Second Moment (ASM): This feature represents the sum of squared elements in the GLCM matrix and reflects the overall uniformity or homogeneity of the image. A higher ASM value indicates a more homogeneous texture, where the pixel pairs are distributed more evenly across the image. Command: skimage.feature.greycoprops(GLCM, ‘ASM’). Formula:

(3) $$\begin{array}{c} \mathrm{ASM}=\sum_{i,j}{\left(\mathrm{GLCM}\left(i,j\right)\right)}^{2} \end{array}$$

4. Homogeneity: This feature measures the closeness of the distribution of elements in the GLCM matrix to the diagonal elements, indicating the level of local homogeneity or similarity in an image’s texture. A higher homogeneity value indicates a greater level of similarity between neighbouring pixel pairs in terms of intensity values and spatial relationships. Command: skimage.feature.greycoprops(GLCM, ‘homogeneity’). Formula:

(4) $$\begin{array}{c} \mathrm{Homogeneity}=\sum_{i,j}\frac{\mathrm{GLCM}(i,j)}{1+{\left(i-j\right)}^{2}} \end{array}$$

5. Correlation: This feature measures the linear dependency between pixel intensities in the image. It indicates how correlated the pixels are in a given direction and provides information about the texture’s pattern and organization. A higher value suggests a higher degree of linear correlation between pixel pairs in the image, representing a more organized and patterned texture. Command: skimage.feature.greycoprops(GLCM, ‘correlation’). Formula:

(5) $$\begin{array}{c} \mathrm{Correlation}=\sum_{i,j}\frac{\left(i-\mu_{i}\right)\left(j-\mu_{j}\right)\cdot \mathrm{GLCM}\left(i,j\right)}{\sqrt{\left(\sigma_{i}^{2}\right)\left(\sigma_{j}^{2}\right)}} \end{array}$$

6. Energy: This feature reflects the overall uniformity or homogeneity of the image texture. It is calculated by the square root of the sum of squared elements in the GLCM matrix, indicating the concentration or "energy" of pixel pairs with specific intensity values and spatial relationships. A higher energy value suggests a more uniform and homogeneous texture in the image. Command: skimage.feature.greycoprops(GLCM, ‘energy’). Formula:

(6) $$\begin{array}{c} \mathrm{Energy}=\sqrt{\sum_{i,j}{\left(\mathrm{GLCM}\left(i,j\right)\right)}^{2}} \end{array}$$

## 4. Experimental Analysis

### 4.1. Evaluation Metrics

#### 4.1.1. Qualitative Analysis

t-Distributed Stochastic Neighbor Embedding (t-SNE) [10]: It is a nonlinear dimensionality reduction technique that maps high-dimensional data to a lower-dimensional space while preserving the local and global structure of the data. It models each high-dimensional data point as a probability distribution in the lower-dimensional space and minimizes the divergence between the probability distributions. In our case, the t-SNE method takes the input of the proposed 22 features and converts them to two-dimensional space for each MRI scan. The sklearn.manifold Python library is used to implement t-SNE with the default settings (n_components = 2, perplexity = 30).

Uniform Manifold Approximation and Projection (UMAP) [11]: It is another nonlinear dimension reduction algorithm that focuses on retaining the local structure of the data. In order to map the high-dimensional data to a lower-dimensional space, UMAP creates a topological representation of the data while maintaining the neighbourhood relationships between the data points. UMAP is implemented with the Python umap package with default hyper-parameters (n_components = 2, n_neighbors= 15, min_dist= 0.1, metric = ‘euclidean’). This study’s visual findings are mostly similar for both t-SNE and UMAP. However, the data are more condensed in UMAP and tend to produce more clusters; hence, t-SNE is recommended as the first choice.

#### 4.1.2. Quantitative Analysis

Domain shift distance: The MMD is widely recognized as a prominent metric in DA research to assess the dissimilarities in data distribution between two domains [26]. It can be mathematically defined as the discrepancy between the distributions of domains *a* and *b*, $MMD_{k}^{2}={∥E_{p}\left[\phi \left(x^{a}\right)\right]-E_{q}\left[\phi \left(x^{b}\right)\right]∥}_{H_{k}}^{2}$, where H(k) represents the Reproducing Kernel Hilbert Space equipped with a kernel function *k*. A decrease in the MMD distance between the two domains after DA or harmonization process signifies a reduction in domain shift.

Domain classification accuracy: Consider a scenario where a random number of samples are selected from two distinct domains, each labelled with its corresponding domain. To evaluate the presence of domain shift or dissimilarity, a domain discriminator or classifier is applied to all the samples, aiming to identify the domain from which each sample originates. The classification outcome serves as a measure of domain shift. A high accuracy in classifying the samples based on their domains implies that the two domains exhibit significant differences, indicating a substantial domain shift. It also supports the robustness of the features used to train the classifier. Conversely, the reduction in domain classification accuracy indicates a decrease in domain shift, making it more challenging to distinguish between them. Support Vector Machine (SVM) with a linear kernel and Random Forest (RF) with a 500 estimator size are used in the experiments as domain classifiers. These classifiers follow the implementation of the Python sklearn package with a five-fold cross-validation setup.

### 4.2. Domain Shift in Multi-Center Datasets

**Figure 2:**
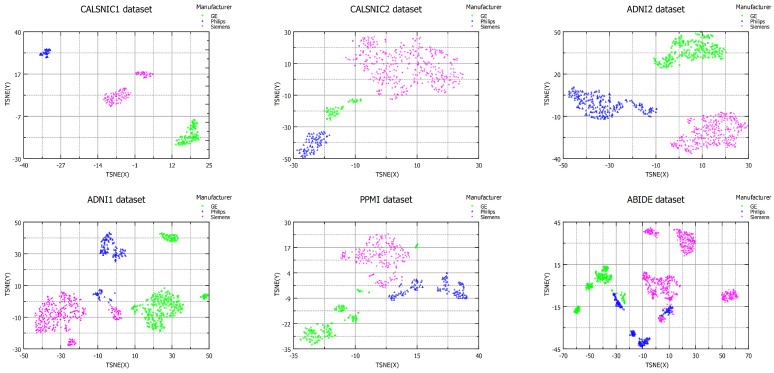
t-SNE plots illustrating data distributions across various datasets: CALSNIC1, CALSNIC2, ADNI2, ADNI1, PPMI, and ABIDE. Each data point in the graph corresponds to an individual MRI scan, using three distinct colours to distinguish scans acquired from different scanner manufacturers.

The multi-center datasets used in this study encompass various factors contributing to domain shift, including scanner manufacturer, model, field strength, image acquisition orientation, resolution, and flip angle. However, when applying the DSMRI framework to these datasets, the resulting clusters primarily demonstrated separation based on the scanner manufacturer parameter, corroborating findings from previous studies [13,14]. Figure 2 presents the visualization of the datasets, considering three distinct domains representing different scanner vendors (e.g., GE, Philips, and Siemens). The first row of Figure 2 depicts the t-SNE plots of the CALSNIC1, CALSNIC2, and ADNI2 datasets, clearly showcasing the separation among data from different manufacturers. In the second row, which pertains to the more challenging ADNI1, PPMI, and ABIDE datasets, some minor overlapping is observed among domains. It might be because of strong similarities in imaging characteristics among those samples. Furthermore, distinct clusters emerge within the same vendor, highlighting the influence of other parameters primarily attributable to the scanner model. These visual findings are supported by the domain shift distance calculated by MMD, as presented in Table 3. Additionally, the domain classification accuracy is consistently around 100% for most cases, which signifies two crucial aspects. Firstly, it highlights the substantial level of domain shift present among the data from different manufacturers, and, secondly, it demonstrates the robustness of the features employed in classifying these domains.

### 4.3. Effects of Scanner Model

**Figure 3:**
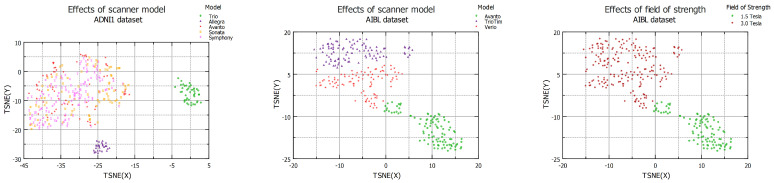
t-SNE plots illustrating the domain shift effects resulting from different scanner models of the same manufacturer, observed in the ADNI1 and AIBL datasets.

This analysis investigates the impact of different scanner models originating from the same manufacturer. A subset of the ADNI1 dataset comprising five Siemens scanner models, namely Trio, Allegra, Avanto, Sonata, and Symphony, is evaluated to understand the effects of different scanner models. The t-SNE plot in the left panel of Figure 3 illustrates that the data from Avanto, Sonata, and Symphony exhibit similarities in their feature space, indicating comparable imaging characteristics. Additionally, it is worth noting that the Trio and Allegra scanners have a magnetic field strength of 3.0 T, while the other three scanners maintain a field strength of 1.5 T. Moving to the AIBL dataset, it consists of data from three different Siemens scanner models: Avanto, TrioTim, and Verio. The middle panel of Figure 3 shows the t-SNE plot for the AIBL dataset, where data clusters closely align with their respective scanner models. Moreover, the right panel of the diagram further confirms the influence of the magnetic field strength as the data with a field strength of 1.5 T are separated from the data with a field strength of 3.0 T in the t-SNE map. Lastly, Table 4 provides information on the domain shift distance and classification accuracy among the different scanner models, offering insights into the variations between these models.

### 4.4. Effects of Resolution

**Figure 4:**
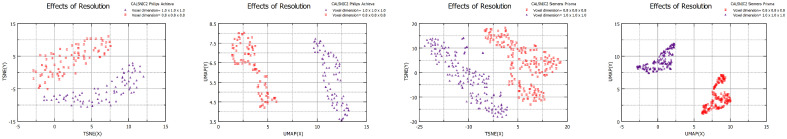
t-SNE and UMAP plots depicting the domain shift effects arising from varying resolutions within the CALSNIC2 dataset.

Within the CALSNIC2 dataset, a total of 86 participants underwent scanning using the Philips Achieva scanner, while 172 participants were scanned using the Siemens Prisma scanner. Notably, the dataset provided two different image resolutions for these participant groups, keeping all other image acquisition parameters constant. Specifically, images with a resolution of 1×1×1 mm^3^ are categorized as low-resolution, while images with a resolution of 0.8×0.8×0.8 mm^3^ are classified as high-resolution in this study. Figure 4 visually demonstrates the similar extent of domain shift observed between the two versions of the images, as reflected in the t-SNE and UMAP plots. Additionally, Table 5 presents the domain classification accuracy, which remains consistently at 100%, along with the corresponding domain shift distance information, further supporting the presence of domain shift.

### 4.5. Effects of T2-Weighted and FLAIR Images

**Figure 5:**
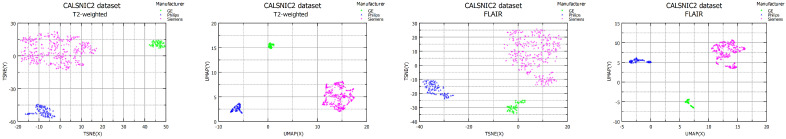
t-SNE and UMAP plots illustrating the domain shift effects observed within the CALSNIC2 dataset due to the utilization of T2-weighted and FLAIR images.

This experiment validates the proposed framework’s effectiveness when applied to T2-weighted and FLAIR images. Within the CALSNIC2 dataset, both FLAIR and T2-weighted images were available for the same population. T2-weighted images offer excellent contrast for evaluating pathologies like inflammation, edema, and fluid-filled structures. On the other hand, FLAIR imaging, a variation of T2-weighted imaging, nullifies the signal from fluids like cerebrospinal fluid (CSF) and enhances the visibility of lesions, particularly those adjacent to CSF-filled spaces. Figure 5 showcases the t-SNE and UMAP plots for the data derived from these two MRI modalities. Interestingly, the clusters representing different manufacturers are even more distinct for these two modalities compared to T1-weighted images. Table 6, presenting the domain shift distance and high domain classification accuracy, provides robust evidence supporting the existence of domain shift in the T2-weighted and FLAIR data while demonstrating the effectiveness of the proposed features.

### 4.6. Effects of Processed Data

**Figure 6:**
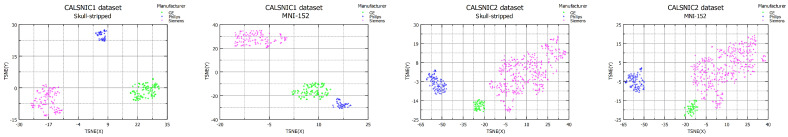
t-SNE plots for the CALSNIC1 and CALSNIC2 datasets showing the effects of data after performing skull stripping and registration to MNI-152 template.

In this experiment, our objective is to evaluate the performance of the data after applying commonly used preprocessing neuroimaging pipelines to the CALSNIC1 and CALSNIC2 datasets. As a crucial step in the preprocessing pipeline, we first utilize the FreeSurfer [40] program for skull stripping. Subsequently, we employ the FSL software [41] to register the MRI scans to the MNI-152 space, ensuring the standardized image and voxel dimensions across all scans. Following these preprocessing steps, we generate t-SNE diagrams to visualize the processed data, as depicted in Figure 6. The visualizations reveal that domain shift remains prevalent in the dataset despite the application of preprocessing techniques. To further confirm the presence of domain shift, Table 7 presents the domain shift distance between pairs of domains, along with a domain classification accuracy of nearly 100%. These findings provide evidence of the substantial impact of domain shift within the dataset, emphasizing the robustness of the proposed features, which consistently demonstrate their efficacy even with the processed data.

### 4.7. Feature Importance

**Figure 7:**
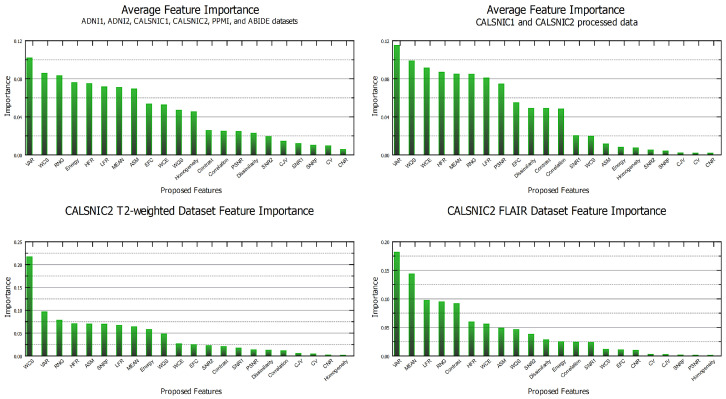
Feature importance ranking across various datasets and data types, assessing domain shift presence through prioritizing the 22 proposed features.

This section examines the significance of different proposed features across various datasets and data types. To accomplish this, we employ an RF classifier and extract the feature importance ranking from the model. The ranking of the features is presented in Figure 7, where the upper left panel displays the average scores of six large datasets utilized in the study. Similarly, the upper right panel depicts the results obtained from the average scores of the processed data from the CALSNIC2 dataset. Interestingly, the ‘VAR’ feature consistently achieves the highest ranking in both cases. The frequency domain features, namely ‘HFR’ and ‘LFR,’ demonstrate notable importance, while the spatial domain features, such as ‘RNG,’ ‘MEAN,’ and ‘EFC,’ also exhibit promising significance. The wavelet and texture domain features mostly occupy the middle area of the ranking chart. Furthermore, the bottom left and right panels illustrate the outcomes obtained from the CALSNIC2 T2-weighted and FLAIR image datasets, respectively. In both cases, features such as ‘VAR,’ ‘RNG,’ ‘MEAN,’ ‘HFR,’ ‘LFR,’ ‘ASM,’ and ‘WQS’ secure positions in the top 10 of the ranking, emphasizing their consistent importance across different data types.

### 4.8. Comparison

**Figure 8:**
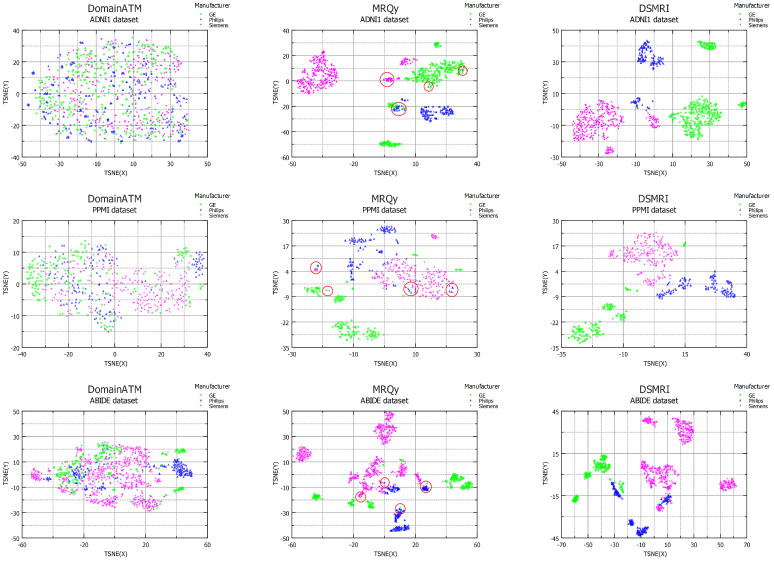
Comparison of the proposed framework with two prior approaches visualizing data distribution through t-SNE plots for the challenging ADNI1, PPMI, and ABIDE datasets.

The comparative evaluation of the proposed DSMRI framework involves two related methods, namely DomainATM and MRQy, with a focus on visualizing the data using t-SNE plots. Figure 8 illustrates the comparison results for three large-scale challenging datasets (e.g., ADNI1, PPMI, and ABIDE). The first column displays the outcomes obtained from DomainATM, revealing inferior performance in clustering the three dominant domains. This can be attributed to the fact that the features utilized by DomainATM, which are the grey matter volumes of different ROIs, do not exhibit a strong correlation with domain shift measurement. Moving to the middle column, the t-SNE diagram generated by MRQy demonstrates a significant improvement in grouping the data based on scanner vendor. However, upon closer observation (shown in red circles), it becomes apparent that a noticeable amount of data either adopts unexpected positions or slightly deviates from the main clusters, suggesting the presence of weaknesses in their features. Finally, in the last column, the proposed DSMRI approach demonstrates a significant superiority over both DomainATM and MRQy in accurately clustering data from different manufacturers or domains. This compelling performance highlights the strength of the features introduced by DSMRI, which exhibit strong correlations with quantifying the degree of domain shift.

## 5. Discussion

The experimental evaluations conducted in this study encompass diverse large-scale datasets that include different patient cohorts spanning a wide range of ages and multiple MRI modalities. These datasets exhibit a multitude of variations in scanner and protocol parameters, including scanner vendor, model, field strength, flip angle, acquisition orientation, resolution, coil configuration, and so on. Understanding the effects of domain shift caused by these individual factors is both intriguing and essential, provided that the remaining parameters remain constant. However, accessing a dataset that provides such a controlled setup is extremely challenging.

Fortunately, the AIBL dataset offers an arrangement that allows for the analysis of three different scanner models from Siemens, thereby examining their effects on domain shift. Additionally, the CALSNIC2 dataset provides an opportunity to analyze the impact of different spatial resolutions while maintaining the homogeneity of other parameters for the same subjects. Furthermore, the CALSNIC2 enables the evaluation and comparison of the performance of T1-weighted, T2-weighted, and FLAIR images for the same population.

Neuroscience researchers often apply common preprocessing steps, such as skull stripping and registration, expecting these procedures to mitigate domain shift or scanner bias issues. However, our study reveals that a similar extent of domain shift persists even after applying skull stripping and registration to the MNI-152 template for the same participants in the CALSNIC2 dataset.

The domain classification accuracy, evaluated using two commonly employed classic classifiers, consistently achieved high accuracy rates across various experiments, reaching nearly 100% in most cases. This finding indicates that the features proposed in this study are robust and meaningful in effectively distinguishing different domains. In contrast, the DomainATM method reported a domain classification accuracy of only 65% in that paper when classifying data from two scanners in a small subset of the ABIDE dataset. This result demonstrates that the grey matter volume features utilized by DomainATM are ineffective in the context of addressing the issue of domain shift.

The proposed frequency domain features, specifically ’HFR’ and ’LFR,’ demonstrated substantial importance in the feature ranking charts, emphasizing their significant contribution to quantifying domain shift. Likewise, the wavelet domain features, namely ’WQS,’ ’WCE,’ and ’WCS,’ played a crucial role in assessing domain shift. We conducted experiments using various wavelet types, including Haar, Daubechies, Discrete Meyer, Symlets, and Coiflets, for wavelet decomposition. The results revealed a high degree of similarity among these wavelet types. However, based on empirical analysis, the Coiflets wavelet type is recommended as the preferred choice in the proposed framework. In the ranking charts, the texture domain features mainly occupied the middle area, signifying their moderate influence on domain shift. Conversely, the noise-related features were predominantly found at lower rankings, indicating that the datasets used in this study were adequately processed and free from noise artifacts.

The framework serves as a valuable QC tool, enabling the assessment of MR image datasets. For instance, the presence of noise artifacts can be identified by the lower values of ’PSNR’ or ’CNR,’ indicating the need for denoising prior to analysis. Similarly, higher values of ’EFC’ or ’CJV’ suggest the presence of motion or shading artifacts in the dataset or specific samples. This information can assist radiologists or experts in making informed decisions regarding the inclusion or exclusion of data before performing computational analysis.

## 6. Conclusions

In the field of neuroscience research, multi-center neuroimaging studies require robust, efficient, and reliable techniques to address the non-biological sources of data variation. ML-based approaches often yield inconsistent results when dealing with data acquired from different MRI scanner models and scanning protocols. This study makes a significant contribution by presenting a simple yet effective unsupervised framework for quantifying the degree of domain shift. After examining a wide range of large multi-center MRI datasets, this study explores the impacts of different scanner manufacturers, models, field strengths, and resolutions in the context of domain shift. Furthermore, the proposed framework demonstrates its adeptness in identifying domain shift, not only in preprocessed MRI data but also across T2-weighted and FLAIR modalities. The findings of this study have important implications for advancing the field of medical imaging and enabling more reliable analysis of multi-center MRI datasets. Moreover, DA and harmonization methods can utilize the proposed framework to validate the effectiveness of their approaches in reducing or eliminating domain shift. Future experiments could explore the application of the DSMRI to more advanced modalities, such as functional MRI (fMRI) and diffusion-weighted images. Such expansion could reveal its versatility and novel advancements in the broader spectrum of neuroimaging research.

## Figures and Tables

**Table 1:** Demographic details of the ADNI1, ADNI2, AIBL, PPMI, ABIDE, CALSNIC1, and CALSNIC2 datasets.

Dataset (#Total)	Group	MRI Scanner Manufacturer					
		GE		Siemens		Philips	
		Sex	Age	Sex	Age	Sex	Age
		(M/F)	(Mean ± Std)	(M/F)	(Mean ± Std)	(M/F)	(Mean ± Std)
ADNI1	AD	85/85	75.5 ± 7.7	85/85	75.0 ± 7.2	43/33	75.7 ± 7.0
(900)	HC	85/85	75.1 ± 5.7	85/85	75.9 ± 5.9	90/54	75.4 ± 5.2
ADNI2	AD	61/40	75.0 ± 8.5	90/57	75.1 ± 7.8	45/58	74.5 ± 7.3
(844)	HC	64/90	74.3 ± 5.9	92/88	74.0 ± 6.4	68/91	75.6 ± 6.4
AIBL	AD	-	-	28/45	73.6 ± 8.0	-	-
(300)	HC	-	-	107/120	72.9 ± 6.6	-	-
PPMI	PD	82/37	61.6 ± 9.7	78/46	63.0 ± 9.8	68/37	61.6 ± 9.9
(520)	HC	17/17	59.6 ± 13.3	71/34	59.6 ± 10.5	20/13	59.7 ± 11.2
ABIDE	ASD	83/15	12.8 ± 2.6	280/40	16.8 ± 8.2	79/7	18.6 ± 9.7
(1060)	HC	91/27	13.9 ± 3.6	275/55	17.1 ± 7.8	94/14	17.6 ± 8.4
CALSNIC1	ALS	21/25	57.0 ± 11.4	43/28	59.6 ± 10.8	17/1	58.1 ± 9.0
(281)	HC	23/33	50.5 ± 11.9	38/28	57.2 ± 8.1	6/18	53.1 ± 8.4
CALSNIC2	ALS	14/4	54.0 ± 11.8	124/65	60.1 ± 10.2	29/19	62.4 ± 8.2
(545)	HC	18/13	60.1 ± 8.8	120/101	54.9 ± 10.5	10/28	61.7 ± 10.8

**Table 2:** Summary of the proposed features used in our study to quantify the degree of domain shift (GLCM = grey-level co-occurrence matrix; SD = standard deviation).

Type	Metric	Description
Spatial domain	MEAN	Mean intensity of the foreground.
	RNG	Intensity range of the foreground.
	VAR	Intensity variance of the foreground.
	CV	Coefficient of Variation to detect shadowing and inhomogeneity artifacts [32].
	PSNR	Peak Signal-to-Noise Ratio of the foreground.
	SNR1	Signal to Noise Ratio of foreground SD and background SD [25].
	SNR2	Signal-to-Noise Ratio of foreground patch mean and background SD [17].
	CNR	Contrast to Noise Ratio to detect shadowing and noise artifacts [33]. Higher values indicate better quality.
	CJV	Coefficient of Joint Variation between the foreground and background to detect aliasing and inhomogeneity artifacts [34]. Higher values also indicate heavy head motion.
	EFC	Entropy Focus Criterion to detect motion artifacts. An indication of ghosting and blurring induced by head motion [17]. Lower values indicate better quality.
Frequency domain	SNRF	Signal-to-Noise Ratio in the Frequency domain, which can be calculated by taking the ratio of the power in the signal to the power in the noise.
	LFR	Low Frequency Response, which measures the ability of the MRI scan to capture low- frequency information in the image.
	HFR	High Frequency Response, which measures the ability of the MRI scan to capture high- frequency information in the image.
Wavelet domain	WCS	Wavelet Coefficient Sparsity measures the amount of sparse information in the wavelet coefficients, which can indicate the presence of artifacts or inhomogeneities.
	WQS	Wavelet-based Quality Score uses the wavelet transform to analyze the spatial frequency content of the image and calculates a quality score based on the magnitude and phase of the wavelet coefficients.
	WCE	Wavelet Coefficient Energy measures the amount of energy present in the wavelet coefficients, which can indicate the presence of artifacts or inhomogeneities.
Texture domain	Contrast	Measures the local intensity variations between neighboring pixels. High contrast values indicate large intensity differences, while low indicate more uniform regions.
	Dissimilarity	Calculates the average absolute difference between the pixel intensities in the GLCM. It quantifies the amount of local variation in the texture.
	ASM	Angular Second Moment measures the uniformity of the intensity distribution in the image and is often used to describe the texture of the tissue.
	Homogeneity	Measures the closeness of the distribution of elements in the GLCM matrix to the diagonal elements, indicating the level of local homogeneity.
	Correlation	Represents the linear dependency between pixel intensities in the image and measures how correlated the pixels are in a given direction.
	Energy	Reflects the overall uniformity in the image. It is calculated as the sum of the squared elements in the GLCM.

**Table 3:** Domain shift distance in terms of MMD and domain classification accuracy for the ADNI1, ADNI2, PPMI, ABIDE, CALSNIC1, and CALSNIC2 datasets.

Dataset	Domain Shift Distance			Domain Classification Accuracy
	GE vs. Siemens	GE vs. Philips	Philips vs. Siemens	GE vs. Siemens vs. Philips
ADNI1	2.03	0.99	3.01	SVM = 0.99 RF = 1.00
ADNI2	18.06	4.31	7.72	SVM = 0.95 RF = 1.00
CALSNIC1	31.60	369.34	105.59	SVM = 0.99 RF = 1.00
CALSNIC2	3.79	2.23	9.97	SVM = 0.99 RF = 0.99
PPMI	1.35	2.02	1.19	SVM = 0.91 RF = 0.98
ABIDE	2.68	1.78	2.30	SVM = 0.93 RF = 0.99

**Table 4:** Domain shift distance and domain classification accuracy for the ADNI1 (Model 1 = Allegra, Model 2 = Trio, Model 3 = Symphony+Avanto+Sonata) and AIBL (Model 1 = Avanto, Model 2 = TrioTim, Model 3 = Verio) datasets to show the effects of various scanner models.

Dataset	Domain Shift Distance			Domain Classification Accuracy
	Model 1 vs. Model 2	Model 1 vs. Model 3	Model 2 vs. Model 3	Model 1 vs. Model 2 vs. Model 3
ADNI1	4.82	1.80	6.82	SVM = 0.99 RF = 1.00
AIBL	5.02	2.62	0.92	SVM = 0.97 RF = 0.98

**Table 5:** Domain shift distance in terms of MMD and domain classification accuracy for the CALSNIC2 dataset showing the effects of different resolutions.

Dataset	Domain Shift Distance Low Resolution vs. High Resolution	Domain Classification Accuracy Low Resolution vs. High Resolution
CALSNIC2 Philips	7.38	SVM = 1.00 RF = 1.00
CALSNIC2 Siemens	8.27	SVM = 1.00 RF = 1.00

**Table 6:** Domain shift distance in terms of MMD and domain classification accuracy for the CALSNIC2 dataset showing the effects of using T2-weighted and FLAIR images.

Dataset	Domain Shift Distance			Domain Classification Accuracy
	GE vs. Siemens	GE vs. Philips	Philips vs. Siemens	GE vs. Siemens vs. Philips
CALSNIC2 T2-weighted	143.75	203.98	130.39	SVM = 1.00 RF = 1.00
CALSNIC2 FLAIR	9.57	6.08	41.73	SVM = 0.98 RF = 0.99

**Table 7:** Domain shift distance in terms of MMD and domain classification accuracy for the CALSNIC1 and CALSNIC2 datasets showing the effects of data after performing skull stripping and registration to MNI-152 template.

Dataset	Domain Shift Distance			Domain Classification Accuracy
	GE vs. Siemens	GE vs. Philips	Philips vs. Siemens	GE vs. Siemens vs. Philips
CALSNIC1 Skull-stripped	37.86	13.29	150.25	SVM = 1.00 RF = 1.00
CALSNIC1 MNI-152	53.97	3.54	250.46	SVM = 1.00 RF = 1.00
CALSNIC2 Skull-stripped	7.88	5.90	39.92	SVM = 0.99 RF = 0.99
CALSNIC2 MNI-152	4.16	6.21	77.24	SVM = 0.98 RF = 0.98

## Data Availability

ADNI1, ADNI2, AIBL, PPMI, and ABIDE neuroimaging data used in the preparation of this article were collected from the ADNI portal (http://adni.loni.usc.edu/) (accessed on 15 May 2023) through a standard application process. The CALSNIC data used in the analysis are available from kalra@ualberta.ca upon reasonable request, following the University of Alberta Ethics Committee’s data sharing and privacy rules.

## Supplementary Materials

The following supporting information can be downloaded at: https://www.mdpi.com/article/10.3390/diagnostics13182947/s1, Table S1: Scanning protocol details of the ADNI1, ADNI2, AIBL, PPMI, ABIDE, CALSNIC1, and CALSNIC2 datasets.
