# Supplementary material for: DSMRI: Domain Shift Analyzer for Multi-Center MRI Datasets
Source: Diagnostics (Basel). 2023 Sep 14;13(18):2947. doi: 10.3390/diagnostics13182947 (PMC10527875; doi:10.3390/diagnostics13182947)
Supplement: Supplementary file 1 [file diagnostics-13-02947-s001.zip › diagnostics-2487587-supplementary.pdf]

Article

DSMRI: Domain Shift analyzer for multi-center MRI datasets Supplementary materials

Rafsanjany Kushol [1,*], Alan H. Wilman [2], Sanjay Kalra [1,3] and Yee-Hong Yang [1]

[1] Department of Computing Science, University of Alberta, Edmonton, AB, T6G 2R3, Canada;
[2] Departments of Radiology and Diagnostic Imaging and Biomedical Engineering, University of Alberta, Edmonton, AB, T6G 2R3, Canada;
[3] Division of Neurology, Department of Medicine, University of Alberta, Edmonton, AB, T6G 2R3, Canada
* Correspondence: kushol@ualberta.ca

Table S1. Scanning protocol details of the ADNI1, ADNI2, AIBL, PPMI, ABIDE, CALSNIC1, and CALSNIC2 datasets.

Dataset	Scanning Protocol	MRI Scanner Manufacturer		
		GE	Siemens	Philips
ADNI1	Model	Genesis Signa, Signa Excite, Signa HDx	Symphony, Sonata, TrioTim, Trio, Avanto, Allegra	Intera Achieva, Intera, Achieva, Gyroscan Intera
	Field Strength	1.5 T / 3.0 T	1.5 T / 3.0 T	1.5 T / 3.0 T
	Flip Angle	8 °	8 ° / 9 °	8 °
	Resolution (mm^3)	1.0 × 1.0 × 1.2 / 0.94 × 0.94 × 1.2	1.0 × 1.0 × 1.2 / 1.25 × 1.25 × 1.2	1.0 × 1.0 × 1.2 / 0.94 × 0.94 × 1.2
ADNI2	Model	Signa HDxt, Signa HDx, Signa Excite, Discovery MR750	Symphony, Skyra, Verio, Avanto, TrioTim	Achieva dStream, Intera, Achieva, Ingenia, Ingenuity
	Field Strength	3.0 T	3.0 T	3.0 T
	Flip Angle	11 °	9 °	9 °
	Resolution	1.05 × 1.05 × 1.2	1.05 × 1.05 × 1.2	1.05 × 1.05 × 1.2
AIBL	Model	-	Avanto, TrioTim, Verio	-
	Field Strength	-	1.5 T / 3.0 T	-
	Flip Angle	-	9 °	-
	Resolution	-	1.0 × 1.0 × 1.2	-
PPMI	Model	Signa HDxt, Genesis Signa, Signa Architect, Signa Excite, Discovery MR750, Discovery MR750w	Symphony, Skyra, TrioTim, Prisma, Verio, Espree, Prisma Fit	Achieva dStream, Achieva, Intera, Gyroscan NT
	Field Strength	1.5 T / 3.0 T	1.5 T / 3.0 T	1.5 T / 3.0 T
	Flip Angle	8 ° / 11 ° / 13 ° / 15 °	8 ° / 9 ° / 15 °	8 ° / 9 °
	Resolution	1.0 × 1.0 × 1.0 / 0.94 × 0.94 × 1.2 / 0.94 × 0.94 × 0.7	1.0 × 1.0 × 1.0 / 1.25 × 1.25 × 1.3 / 0.49 × 0.49 × 2.0	1.0 × 1.0 × 1.0 / 0.94 × 0.94 × 1.2 / 1.0 × 1.0 × 1.2
ABIDE	Model	Signa Discovery MR750	Allegra, Verio, TrioTim, Prisma,	Achieva, Intera
	Field Strength	3.0 T	3.0 T	3.0 T
	Flip Angle	8 ° / 15 °	7 ° / 8 ° / 9 ° / 10 °	7 ° / 8 °
	Resolution	1.0 × 1.0 × 1.0 / 0.86 × 0.86 × 1.5 / 1.02 × 1.02 × 1.2	1.0 × 1.0 × 1.0 / 1.0 × 1.0 × 1.33 / 0.5 × 0.5 × 1.2	1.0 × 1.0 × 1.0 / 0.98 × 0.98 × 1.2 /
CALSNIC1	Model	Discovery MR750	Prisma, TrioTim	Intera
	Field Strength	3.0 T	3.0 T	3.0 T
	Flip Angle	11 °	8 °	9 °
	Resolution	1.0 × 1.0 × 1.0	1.0 × 1.0 × 1.0	1.0 × 1.0 × 1.0
CALSNIC2	Model	Discovery MR750	Prisma, TrioTim	Achieva
	Field Strength	3.0 T	3.0 T	3.0 T
	Flip Angle	16 °	10 °	10 °
	Resolution	1.0 × 1.0 × 1.0	1.0 × 1.0 × 1.0	1.0 × 1.0 × 1.0
